# Medical Items and News of Interest to the Physician

**Published:** 1881-01

**Authors:** 


### For the Use of Physicians.

CULLED FROM THE STANDARD MEDICAL JOURNALS OF THE WORLD.

**Jamaica Dogwood.**

Dr F. H. Little, in the *Therapeutic Gazette*, recommends the fluid extract of this drug as a substitute for opium. Taken in from one-half to one drachm doses, he declares it never fails to relieve pain.

**Sore Nipples.**

Dr. Howell recommends the following in the Canada Medical Record:—

℞

Tannin, ʒ j.

Sub-nit bismuth, ʒ ij.

Vaseline, ℥ j.

M. Sig. To be applied constantly when the child is not nursing.

**Balsam Copaiba Emulsion.**

Balsam of copaiba,

Powdered acacia, āā ʒ iij.

Simple syrup, ʒ vi.

Water, ℥ vj.

Mix the powder with the balsam, and add six drachms of water, stir until the emulsion is formed, then gradually add the remainder of the water and syrup.

**Asthma.**

Dr. A. W. M. Robson finds nitroglycerine suitable for such cases as are usually proper for the use of nitrate of amyl, but it is more permanent in its effects and more powerful. One or two drops of a *one per-cent* solution will usually suffice, although sometimes three or four minims may be needed. One to eight minims have proved servicable in angina pectoris, the dose being gradually increased to the larger amount.

**Rectal Alimentation.**

In a case of severe and persistent vomiting at Bellevue Hospital (*Medical Record*, July 31st,) the patient was nourished very satisfactorily with enemata administered every three hours, composed as follows:

Milk, ℥ ij.; half an egg; whiskey, ℥ ij., and McMunn's elixir of opium, gtt. x. The vomiting and pain were at once relieved. Two days previous to taking this note defibrinated blood was substituted for the above mixture, and ℥ iij., every three hours, were employed with satisfactory results.

**Toothache.**

Dr. Sporer, of St. Petersburg, uses chloral dydrate in the following manner:

Take three or four small lumps of chloral, wrap them in a little wadding, place this tampon in the hole in the tooth, and let it remain until dissolved. The most severe toothache will disappear in a few minutes under this treatment.

**Hemorrhoids.**

In cases of hemorrhoidal congestion, Vidal regards Capsicum Annum as the best remedy.

He prescribes four or five pills daily, each containing 20 centigrams, half at breakfast time and half at supper time. Under this influence the congestion and all the painful symptoms which accompany it disappear rapidly.—*Journal de Medicine.*

**Chian Turpentine Emulsion**

Chian turpentine, ʒij.

Ether, ʒ iv.

Powdered acacia, ʒ ij

Water, ℥ vj.

Dissolve the turpentine in the ether, and filter, washing the filter with a drachm of ether, mix well with the gum and add one-half ounce of water, stirring till the emulsion is perfect, lastly add the remaining water.

**Administration of Iodide of Potassium.**

A most palatable way of administering the iodide to children, or to adults whose gastric membrane is easily irritated, is as follows in any dose required:

℞

Potass. iodidi, gr. x.
Syrupi cydonii, ℥ j.

M. Sig. In a glass of water.

I prepare the syrup of cydonium as follows:

℞

Cydonium (quince seeds) 1 part.
Cydonium malum (quince fruit) 3 parts.
Simple syrup, 4 parts.
Water sufficient.

Boil together until seeds and fruit can be crushed with a spoon, then boil until the mass becomes of the consistance of molasses. Strain through a fine sieve. The syrup prepared this way keeps as long as jelly. It is an elegant vehicle on account of its delicious flavor and its mucilaginous properties, giving us in one preparation a demulcent as well as a placebo.

I commend this vehicle not for the iodide alone, but likewise for the nitrate of potassa, and such drugs as are objectionable when given in a watery solution.—*Med. Herald.*

**Camphor and Chloral.**

Dr. Simmons has used a mixture of equal parts, in doses of twenty drops, in mania with sleeplessness, with the effect of causing prolonged and refreshing rest. In mania delirum tremens, etc., he has found it to serve as a sedative when other remedies of this class have failed. The effect of combination is far in excess of that produced by either remedy given separately, and Dr. S. mentions a case in which prolonged narcotism for several days followed the accidental taking of a dose of two drachms.—*N. Y. Med. Jour.*

---

**Diphtheria.**

Dr. Perate has seen very favorable results in diphtheria, after the use of the combination of camphor and carbonic acid, recommended by Soulez:

9 gm. of carbolic acid are mixed with 25 gm. of camphor and 1 gm. of alcohol, and the whole mixed with 35 gm. of fixed oil of almonds. The affected parts are brushed over with this solution, throughout their whole extent, every two hours in the day and every three hours at night. After a few days the applications are renewed at greater intervals. The mixture has a very disagreeable taste, but children soon get used to it.

---

**Digestive Derangements.**

According to Niewodniczanki, sulphate of zinc may be advantageously employed in place of nitrate of silver in affections of the digestive organs (*La Press Medicale Belge*,) The sulphate of zinc is less easily decomposed, is better tolerated and seems very active. It has been employed with success in acute and chronic affections of the stomach in gastralgia; the addition of codeia or opium to the sulphate of zinc is unnecessary, for the salt is itself calmative. It is given in doses of 6 to 10 centigrammes a day. It is also useful in diarrhœa.

---

**Diphtheria.**

M. Vidal advocates the employment of tartaric acid in diphtheria (*La France Medicale.*) Local action on the false membrane is necessary because they have a great tendency to propagation by a sort of auto-inoculation comparable to that which takes place in certain skin diseases. The formula he employs is this—Tartaric acid, 10 grammes; glycerine, 15 grammes; distilled mint water, 25 grammes. The tartaric acid acts on the false membrane, which it changes into a gelatinous mass and favors its expulsion. Applications of it should be made about every three hours, and should be followed a short time after by applications of lemon juice.

---

**Poisoning.**

Mr. Sonneberg has found sulphate of sodium to be useful in the treatment of poisonous effects sometimes caused by the prolonged use of dressing with a 5 per cent. solution of carbolic acid. The dose is 5 to 8 gm. of the sulphate for adults, and 2 to 5 gm. for children, in 200 gm. of water. The urine which in such cases has become deep green tinged with brown, quickly reassumes its normal color, and the dressings can then be resumed without danger.—*Zeitsch d. Oest. Apoth.*

---

**Vehicle for Salicylic Acid.**

A pleasant and agreeable method of administering salicylic acid is as follows: Take of Oswego corn-starch one tablespoonful, to be thoroughly rubbed up in several ounces of cold water. Add a quart of milk, set on the fire, and stir until the mixture has boiled sufficiently to become homogeneous. The addition of sugar and essence of vanilla or lemon will give a delicious blanc-mange. Twenty grains of the salicylic acid can be rubbed up in a mortar with a cupful of the blanc-mange, which may be eaten warm or cold. The acid taste is entirely disguised, and a medicine irritating to a healthy stomach can be safely administered in combination with a nutritious but light food to such patients as are in need thereof—*Louisville News.*

---

**Nitro-glycerine.**

Nitro-glycerine can be prepared for use in a solution either of alcohol or ether, a one-per-cent-solution in alcohol being that generally preferred. It being also soluble in melted cocoa butter, this has been employed for making it into pills, but in this form it does not act so quickly as in alcoholic solution. A better plan than this is to add an equal quantity of chocolate paste to this pill mass, and then make it up into lozengers of any desired size, which it would not be disagreeable to chew up in the mouth, and thus gain rapid absorption. Nitro-glycerine bids fair to prove an anti-neuralgic of the greatest value, especially in cases of angina pectoris and such other affections as nitrite of amyl has hitherto been much used in, taken at intervals of two or four hours, in doses of about two drops of the one per cent. solution, and increased until full physiological action is obtained. The relaxed condition of the blood-vessels induced lasts usually about half an hour. Doses of the above strength and frequency have been continued through several consecutive days with safety and success in warding off threatening attacks of angina pectoris in cases in London hospitals.—*Louisville Medical News.*

**Itch.**

In the Medical News and Abstract, June 1880, Prof. Hardy is represented as having employed for eighteen years, in the treatment of itch, an ointment of lard, one part ; flowers of sulpher, one-sixth part ; sub-carbonate of soda, one-twelfth part. Thorough and almost violent frictions of this ointment, continued for twenty or thirty minutes, especially at the natural bends and folds of the skin, always cure. The friction was repeated only once in four or five thousand cases, and all the patients were cured. After rubbing, the ointment should remain all night. On the following day an emollient bath may be taken. This may be repeated daily for a week.—*Chicago Med. Review.*

**Castor-oil Emulsion.**

The following is recommended by Mr. Charles A. Heinitsh, of Lancaster, Pa., who states that it contains 50 per cent. of oil, is palatable, does not gripe, and given in the dose of an ounce, is nearly or quite as effective as the same quantity of oil:

℞

Ol. ricini, fl. ℥ viij.

Glycerini, fl. ℥ ij.

Syr. simplicis, fl. ℥ iij.

Ol. cinnamomi, ♏ x.

Ol. menthæ viridis, fl. ʒ ss.

Aq. menthæ viridis, fl. ʒ iij.

To make O. i. of emulsion. The stock bottle containing it should be kept in a cool cellar, but the amount required for dispensing will keep well in the store.—*Trans. Penn. Pharm. Assoc.*

**Onions in Consumption.**

Dr. William H. Pearse, physician to the Plymouth Public Dispensary, recommends the free use of onions for consumptive patients. He says: "I have in a former paper mentioned the frequent desire of phthisical patients for onions, salted and smoked fish, etc. Of those asked, forty had a great desire for onions against eight without such desire. Twenty-six desired pickles and vinegar against four who did not. I cannot avoid again remarking on the frequency with which onions are debarred young delicate people and phthisical patients. It is a continually recurring experience with me to hear young people say how great is their desire for onions, and which are often preferred raw, eaten with a little salt; and it is rarely that I have heard that onions disagree.

"I conceive that it is of the greatest importance to follow Nature's lead in the matter of the appetite. I conceive, further, that a marked passion for a special food, such as that of the phthisical for onions, puts us on a right path toward further knowledge."

**Filling for Teeth.**

A filling that has been very successfully used for teeth, and which is next to gold leaf in permanency, is made of the following ingredients:

Oxide of zinc (recently made by burning) 200 parts.
Powdered silica, 8 parts.
" borax, 4 parts.
" glass, 5 parts.

Mix and pass through a very fine sieve.

To be kept in a well stoppered bottle. When required for use, a little of the powder is mixed quickly with a concentrated solution of chloride of zinc, so as to make a thick paste, which is pressed into the cavity of the tooth and will harden in less than ten minutes. It forms a hard white cement which will last for years.—*Pharm. J. and Trans.*

**Antiseptic Dressings.**

Dr. Th. Siegen recommends, in the *Deutsch. Clin. Wochensch.*, oil of eucalyptus for the preparation of moist dressings, which are inexpensive and rapidly prepared. 3 grams of oil of eucalyptus are dissolved in 15 gm. of alcohol, and the solution mixed with 150 gm. of water (which, of course, separates most of the oil, though it can be retained in suspension for some time.) This solution is then incorporated into ordinary gauze, previously well washed so that it will absorb the liquid readily. This gauze is applied moist, covered with guttapercha paper and the whole well fastened with gauze-bandages. In cases where thymol-dressings were found to be inert, the eucalyptus-dressing gave very good results. It has also the advantage of not affecting the skin in any way and not producing eczema, even if the strength of the solution is raised to 5 per cent. Much depends on the quality of the oil of eucalyptus, since there are various sorts, and they do not all seem to act alike. The above is taken from the *Pharm. Centralh.*

**Anthelmintic Action of Kameela.**

M. Blondeau has employed the tincture of kameela with success in cases of tænia. He gave, in one case, f ʒ vj. tincture of kameela in infusion of sage, divided into three doses and taken at intervals of an hour—at nine, ten and eleven in the morning. At one o'clock in the afternoon, without having experienced the least colic, the patient voided an enormous tænia, the head of which was unfortunately missing.

This anthelmintic possesses, according to M. Davaine, who also advocates its use, the following advantages; it is not disagreeable to take; it does not cause colic, and it need not be associated with a purgative.

Kameela is obtained from the capsules of one of the Euphorbiacæ—the Rotzleria tinctoria. It is a red powder used in dyeing silk. Its use as an anthelmintic has been suggested by Hunsby. Anderson, who has also employed the tincture, has never gone beyond the dose of f ʒ iijss —*Bull. Gen. de Therap.*

---

**New Method of Arresting Gonorrhœa.**

I read with great pleasure the article headed as above, by Mr. Cheyne, and wish to state that I have adopted his method of passing medicated bougies up the urethra for acute and chronic gonorrhœa. The bougies I used were made by Kirby & Co., 14 Newman Street, Oxford Street. The other day I thought I would use iodoform in the shape of a bougie; I therefore ordered some containing five grains in each, and have been very gratified with the result, which has quite come up to my expectation. I have been in the habit of using iodoform, both in the form of ointment and of powder for some years, and with marked success, in the treatment of indolent varicose ulcer of the leg, soft chancres, etc.

The method I adopt in the treatment of gonorrhœa is this; I first order the patient an injection containing ten minims of liqor plumbi and two grains of sulphate of zinc to an ounce water: to be used frequently, until the acute symptoms have subsided. I then pass a No. 9 bougie up the urethra as far as the ulcerated spot. I then apply a piece of lint over the orifice of the urethra, under the prepuce; and tell him not to pass his urine for some hours afterwards. I order him to take as little liquid as possible, and no stimulants. I generally pass one or two bougies a day. My patients generally get rid of the gonorrhœa in a week. The only constitutional treatment I adopt is a brisk purgative, followed by tonics.—*J. Brindley James, M. R. C. S. Eng., in British Med. Jour.*

---

**Diphtheria.**

Dr. G. W. Garrison, in the Cincinnati *Lancet and Clinic*, advises the following treatment :—

First twenty-four hours: Saline cathartic, Topical : tincture iodine all over the cervical region, paint it every four to six hours. Locally:

℞
Hyposulphite sodæ, ℥ i.
Aq. menth. pip., ℥ vij.
Bro. ammonium, ʒ iv. Mix.

Gargle every two hours and take one teaspoonful at same time.

Constitutional treatment:

℞
Carb. ammonia, ℥ ss.
Acid acet. dil., f ℥ i. Mix.

After effervescing add:

Tinct. ferri chloridi, ℥ i.
Glycerin pure.
Aq. Menth. pip., āā qs.
Ft. oz. viij. (f ℥ viij).
S. One teaspoonful every two hours.

At the expiration of forty-eight hours we use the iron mixture every two hours, alternating with whisky and quinine, and continue the latter until our patient is perfectly restored. Topical applications are of but little consequence after two to three days. In place of the iodine some oleaginous mixture should be applied to the neck for two or three days.

---

**Sweating of the Feet.**

Dr. George Thin has recently made a fruitful investigation of this subject, the report of which is published in the *British Medical Journal* for September 18, 1880, and from which the following is abstracted :

The patient who has afforded me the opportunity of investigating the cause of the smell, is a young woman, aged 22, who has suffered from evil-smelling feet, with soreness of the heels, for several years. Her hands are usually moist, or even wet, but are always odorless. The smell from the feet is not constant, disappearing in dry bracing weather, and reappearing when the weather is moist and depressing.

The first experiment I made was to subject the soles of the stockings and boots to the action of an antiseptic solution. The success was complete, the odor being entirely banished. The antiseptic precautions having been soon neglected, the smell returned, and I took the opportunity of investigating its cause more minutely.

The sole of the stocking, a few hours after it was put on, was found to be quite wet; and a stocking, if worn for a whole day, was so extremely offensive that, when held close to the nostrils, its overpowering fetor was comparable to that of putrid blood. The inside of the boot was equally wet and offensive; but, at the very time that the stocking and boot smelt so strongly, the heel itself, exuding moisture profusely, had no disagreeable odor. The sole of the heel was reddened and tender, and macerated around the the edge, like a washerwoman's palm.

The reaction of the moisture in the stocking and in the sole of the boot was alkaline, that of the moisture exuding from the skin of the sole of the heel faintly alkaline, whilst that of the perspiration of other parts of the body was acid.

The fluid from the sole of the heel was thus shown to be not pure sweat, the faintly alkaline reaction being doubtless due to the serous discharge accompanying the eczema set up by the local hyperidrosis.

The fluid in the sole of the stocking was found to be teeming with bacteria forms, the nature and development of which I have carefully investigated. These investigations have produced results of some scientific interest, which I have communicated to the Royal Society. The rapid development of bacteria in the fluid which exudes from the soles is doubtless favored by the alkaline reaction produced by the mixture of serous exudation with sweat.

The treatment instituted in this case is as simple as it has been effective. The stockings are changed twice daily, and the stocking-feet are placed for some hours in a jar containing a saturated solution of boracic acid. They are then dried, and are fit for wear again if it be desired. The boracic acid effectually destroys the smell. But to kill the bacteria in the stockings is not enough. The leather in the bottom of the boot is wet and sodden, and smells as vilely as the stocking. This difficulty is got over by the use of cork soles. I directed my patient to get half a dozen, which she finds sufficient. A pair must only be worn one day unchanged; at night, they are placed in the boracic jar, and are put aside the next day to dry. If these directions be accurately carried out, the evil smell is perfectly destroyed.

The boracic acid solution is an excellent application to the painful skin in these cases. When the tender skin of the soles is washed with it, a sensation of coolness succeeds the feeling of heat and tension which are the usual accompaniments of the eczematous condition associated with the smell, and the skin becomes harder and loses its abnormal redness.

The bacteric fluid would seem to act as a direct irritant to the skin. My patient assures me that, if she wears stockings which have been dried without being disinfected, irritation is speedily felt; and that the cork soles, if worn a second day without having been purified, act in a similar way.—*Medical News and Abstract.*

---

**Hospital Practice.**

The following formularly are taken from the reports of the New Haven Hospital:—

In cases of *acute rheumatism*, the routine treatment is—

℞ Salicine, gr. xx.

In wafers every two hours, combined with external bathing of the parts with an alkaline wash and wrapping the afflicted joints in cotton batting.

The salicine has been found to answer all the purposes of salicylic acid, and is much less irritating to the stomach, and less likely to produce nausea; and furthermore, is very much cheaper.

As to house prescriptions we have very few, but of them one is used almost constantly as a *stimulent and febrifuge*:

℞

Camphoræ, ℈ j.

Ammon. carb, ʒ j.

Muc. acaciæ, ℥ j.

Aquæ. Oj. M.

Sig.—℥ ss. every two hours.

The *lictus morphiæ* is also much used. The formula for it is as follows:

℞

Morphiæ sulph, gr. j.

Spts. chloroform, ʒ j.

Muc. gm. acaciæ, ℥ j

Syr. tolut. q. s. ut ft. ℥ ijss.

M.

A favorite *diarrhœa mixture* is as follows:

℞
Tr. opii.
Tr. capsici.
Tr. rhei aromat.
Spts. camphoræ.
Spts. menth. pip., āā. part. æq.

We have of late used quite entensively *codeia as a substitute for morphia*, and with very good results. It is given in capsules, and can be taken by those who are unable to take morphia in any form.

